# 

**Published:** 1842-09

**Authors:** 


### WESTERN JOURNAL

OF

### MEDICINE AND SURGERY.

### EDITORS.

---

DANIEL DRAKE, M. D.,

PROFESSOR OF PATHOLOGICAL ANATOMY AND CLINICAL MEDICINE

IN THE MEDICAL INSTITUTE OF LOUISVILLE:

LUNSFORD P. YANDELL, M. D.,

PROFESSOR OF CHEMISTRY AND PHARMACY IN THE SAME:

THOMAS W. COLESCOTT, M. D.

BOOKS RECEIVED.

Our acknowledgements are due to the Committee of Publication for a copy of the revised edition of the *Pharmacopœia of the United States*.

Dr. Paine will also accept our thanks for a copy of his *Materia Medica*, received some time since.

The *Proceedings of the Thirteenth Annual Meeting of the Medical Society of the State of Tennessee* have reached us. Notices of all these will appear in the ensuing number of the Journal. By the way, can our neighbor of the Western Lancet tell us aught of the proceedings of the fifth session of the Medical Convention of Ohio? We thought the transactions were to be published. C.

### RECEIPTS OF THE MEDICAL JOURNAL.

FOR THE MONTHS OF JULY AND AUGUST.

| | |
|---|---|
| Dr. B. L. Hatch, Aberdeen, Miss. | $1 00 |
| D. B. Rice, Oquawka, Ill. - - | 5 00 |
| D. S. Smith, Juliet, Ill. - - - | 5 00 |
| Drs. Tate & Winter, Columbus, Miss. | 5 00 |
| Dr. T. M. Armstrong, Livingston, Miss. | 5 00 |
| S. O. Scruggs, Jones' Bluff, Ala. (dis. $3 00, postage 25 cts.) - - | 10 00 |
| J. M. Robertson, Burlington, Iowa | 5 00 |
| —— Marke, Newark, Ohio . - | 3 00 |
| Geohegan & Helmick, Baltimore, O. | 15 00 |
| W. H. Wisehard, Far West, Ia. - | 5 00 |
| W. Anderson, Laurel, Ia. - - | 5 00 |
| T. R. Beck, Albany, N. Y. - - | 5 00 |
| J. Clayton, Christiansburgh, Ky. - | 5 00 |
| —— Westerfield, Glasgow, Ky. - | 5 00 |
| J. A. Blackmore, Gallatin, Tenn. - | 5 00 |
| W. E. Murphy, Decatur, Ala. - | 10 00 |
| J. C. Harris, Cedar Bluff, Ala. (dis. $1,50) - - - - - - | 3 50 |
| J. H Charlton, Elm Hill, Tenn. - | 5 00 |
| Dr. J. McCullough, Reynoldsburgh, O. | 5 00 |
| D. Adams, Prairieton, Ill. - - | 5 00 |
| J. Cobb, Cumberland Iron Works, Tenn. - - - - - - | 5 00 |
| R. L. Wilkinson, New Haven, Ky. | 5 00 |
| A. D. Cage, Mt. Henry, Tenn. - | 5 00 |
| N. F. Scales, Lewisburgh, Tenn. - | 5 00 |
| W. D. Dorris, Nashville, Tenn. (post. 38 cts.) - - - - - - | 10 00 |
| —— Davis, Murfreesboro', Tenn. | 5 00 |
| J. Redhead, Woodville, Miss. - | 5 00 |
| A. B. Dodd, Bolivar, Miss. - - | 5 00 |
| —— Prosser, Gallipolis, O. - - | 5 00 |
| T. Brandon, Huntsville, Ala. - | 5 00 |
| W. Johnson, Floydsburgh, Ky. - | 5 00 |
| J. M. Riley, Orleans, Ia. - - | 5 00 |
| —— Jewett, Lyons, Mich. - - | 5 00 |
| C. Smith, Toledo, O. - - - | 5 00 |
| Drs. Murphy & Donald, Franklin, Ia. - | 5 00 |
| Baker & Kittredge, Norwalk, O. - | 5 00 |

### DELINQUENT LIST.

It will be seen that we continue our list of delinquents, which a press of matter last month prevented. Next month the list will be completed, and it will behoove all in arrearages, who are not desirous of having their names appear *in print*, to remit the amount of their accounts without delay. Post Masters will frank remittances.

TENNESSEE.

J. Drake, South Carroll
J. J. Gibson, Hampshire
Wilson & Bearden, Petersburgh
L. M. N. Cook, Silver Spring
T. J. Walton, Mulloy's
S. B. Robinson, Versailles
J. M. Dean, Lynchburg
A. F. Clement, Lexington
W. H. Warren, Lexington
W. T. Eatherty, Rural Hill
J. A. Lecky, Ripley
S. Richardson, Rutherford.
W. D. Lee, Rutherford.
W. J. J. Morrow, Chattawogga
J. W. Koen, Colliersville
J. M. M. Cornelius, Germantown
Robinson & Hagan, Carthage
J. B. Copeland, Nolandsville
R. Forbis, Elkton
M. W. O'Brien, Springfield,
W. J. C. Rogers, Murfreesboro'

NEW YORK.

Wm. Welch, Penfield
G. Standing, Utica

ARKANSAS.

W. A. Threlkeld, Helena

J. H. McCarty, Ozark

Dr. Beach, Lawrenceville

J. Eaton, Pine Bluff

LOUISIANA.

B. B. Smith, Shreveport
J. B. Henderson, New Orleans.
J. Davis, Millekin's Bend
T. Ingersoll, Cherryville
E. Ragan, Columbia

ALABAMA.

P. L. Gilbert, Isny, Washington County.
R. L. Graves, Leesville
D. W. Ford, Meridianville
E. M. Hussey, Waterloo
W. R. Davie, Waterloo
W. F. Johnson, Benton
J. Durno, Mobile, or Alton, Ill.
J. T. Reese, Syllacogga.

MISSISSIPPI.

Harris & McLaen, Wahallock
E. F. Bouchelle, Columbus
S. D. Kennedy, Holmesville
D. B. Nailer, Vicksburgh
—— Hogan, Panola
A. P. Reed, Fort Adams
J. A. Hodges, Garlandsville
O. S. Echols, Hamilton
L. Shelby, Warrenton

ILLINOIS.

O. Abbey, Quincy
W. H. Efner, Lacon
J. W. Chenoweth, Auburn
C. L. Duncan, Marshall
J. R. Colwell, Farmington
J. Kirkpatrick, Fox P. O.
W. Allison, Paradise
W. Patton, Oakland
T. J. Beckwell, Rushville
J. Jayne, Carlinsville
B. F. Edwards, Alton
C. F. Falley, York
H. Symmes, Equality
W. W. Higgins, Jones' P. O.
W. O. Chamberlain, Princeton
Wood & Goodwin, Holland's Grove
F. Canterbury, Cole's C. H.
H. P. Griswold, Plymouth
J. M. Metcalf, Winchester
W. Kellar, Decatur

INDIANA.

B. F. Russell, New Washington
J. H. McNutt, Annapolis
D. Long, Blackford
D. Hoover, Wolf Lake
J. L. Plaff, Westfield
F. C. Gale, Vevay
N. B. Odelle, Lafayette
J. M. Bell, Dublin
P. Q. Stryker, Rockville.
W. Anderson, Somerset
W. H. Martin, Rushville
C. West, Richmond
L. Edwards, Greenfield
J. A. Pomeroy, Roseville
E. H. Bruce, Peru
S. P. Wort, Brownstown.
J. Prosser, Orleans
H. Single, Orleans
T. Losey, Laporte
W. Simpkins, Lebanon
W. M. Gentry, Frankfort
W. V. Snyder, Frankfort
R. Hobson, New Harmony
J. H. Herryman, Bowling Green
D. Todd, Monticello
A. Poteet, Monticello
C. Wallace, Indianapolis
S. T. Clark, Russelville
J. W. Porter, Greenville
J. C. Clarke, Greenville
T. Sturges, Fort Wayne
A. D. Sweet, Franklin
E. S. Blue, Leesburgh
J. Darr, New Castle
J. V. Wayman, New Castle
J. W. Crooks, Rockport
J. W. French, Troy
A. M. Murphy, Bloomington
I. N. Griffith, Jeffersonville
D. C. McGee, Mount Pleasant
J. McCallen, McAllen's Cross Roads
J. S. Deputy, Madison
—— Tilton, Madison
S. Wyatt, Lexington
W. W. Goodwin, Charlestown
G. R. McCoy, Charlestown

The Western Journal of Medicine and Surgery is published monthly by the undersigned, at the corner of Main and Fifth streets, Louisville, at $5 per annum, payable in advance. Each number contains from 80 to 84 pages making two volumes in the year of about 500 pages.

Contributions to its pages by the Physicians of the Valley of the Mississippi, addressed to the Editors, are respectfully solicited.

Letters on business, to be addressed, postage paid, to the publishers.

☞Postmasters, by the regulations of the Postoffice Department, will frank letters containing subscription money, and all remittances so franked are at the risk of the publishers.

*August*, 1842. PRENTICE & WEISSINGER.

